# Naringenin Enhances the Inhibitory Effect of Regorafenib on HepG2 Hepatocellular Carcinoma Cell Viability with Modulation of E-Cadherin and MRP1 Gene Expression

**DOI:** 10.3390/ijms27209347

**Published:** 2026-10-21

**Authors:** Sara A. Gaballah, Ahmed I. Abulsoud, Asmaa Saleh, Noha A. Eldesoky

**Affiliations:** 1 Department of Biochemistry & Molecular Biology, Faculty of Pharmacy (Girls), Al-Azhar University, Nasr City, Cairo 11823, Egypt; noha-bio@hotmail.com or noha-bio@azhar.edu.eg; 2 Department of Biochemistry and Molecular Biology, Faculty of Pharmacy (Boys), Al-Azhar University, Nasr City, Cairo 11823, Egypt; ahmed.abulsoud@hu.edu.eg; 3 Department of Biochemistry, Faculty of Pharmacy, Heliopolis University, 3 Cairo-Belbeis Desert Road, El Salam, Cairo 11785, Egypt; 4 Department of Pharmaceutical Sciences, College of Pharmacy, Princess Nourah Bint Abdulrahman University, P.O. Box 84428, Riyadh 11671, Saudi Arabia; asali@pnu.edu.sa

**Keywords:** hepatocellular carcinoma, HepG2 cells, MRP1, E-cadherin, regorafenib, naringenin

## Abstract

Hepatocellular carcinoma (HCC) is a life-threatening malignancy. Although regorafenib is used for HCC treatment, its clinical utility is limited by significant adverse effects. This study evaluated whether combining regorafenib with naringenin could enhance its anticancer activity against HepG2 cells. This study included seven groups of HepG2 cancer cells: an untreated control group, a naringenin-treated group (100 μM), a regorafenib-treated group (1 μM), and four groups receiving naringenin (100 μM) combined with decreasing concentrations of regorafenib (1, 0.75, 0.5, and 0.25 μM). Cell viability and the expression of E-cadherin and MRP1 were assessed. All treatments significantly reduced cell viability compared with the control group, with greater inhibition observed in the combination-treated groups than in the monotherapy groups. Combination treatment also increased E-cadherin expression and decreased MRP1 expression. The combination of regorafenib (1 μM) and naringenin (100 μM) exhibited the greatest growth inhibition, the highest E-cadherin, and the lowest MRP1 expression levels. Notably, combining the IC_50_ of naringenin with one-quarter of the regorafenib IC_50_ significantly enhanced E-cadherin expression and reduced MRP1 expression compared with regorafenib alone. The present findings suggest that naringenin enhances the anticancer effects of regorafenib in HepG2 cells and modulates E-cadherin and MRP1 gene expression.

## 1. Introduction

Hepatocellular carcinoma (HCC) is the third leading cause of cancer-related mortality worldwide. It has high incidence and mortality due to inadequate medications and delayed diagnosis resulting in distant metastasis with poor prognosis [1]. Metastasis is a highly complex and dynamic process characterized by a series of interconnected events, including angiogenesis, epithelial–mesenchymal transition (EMT) and cellular invasion. As cancer cells progress through these stages, they encounter diverse microenvironmental and cellular stresses, with oxidative stress representing a major and persistent challenge that can influence tumor progression and metastatic behavior [2].

Reactive oxygen species (ROS) significantly drive epigenetic remodeling including alterations in DNA methylation, histone modifications, and non-coding RNA pathways, thereby modulating metastasis-related gene networks [2].

Developing novel approaches that can overcome tumor metastasis and achieve greater efficacy is the main goal of the most current HCC research [3,4].

The oral multi-kinase inhibitor (MKI) regorafenib targets a variety of kinases including platelet-derived growth factor receptor (PDGFR), vascular endothelial growth factor receptor (VEGFR), fibroblast growth factor receptor (FGFR), and tyrosine kinase with immunoglobulin and epidermal growth factor homology domain 2 (Tie-2) [5,6].

Naringenin (5,7,4-Trihydroxyflavanone) is a flavanone glycoside formed from naringin. Most citrus fruits, including oranges, lemons, and grapefruit, contain naringenin [7]. Naringenin has several bioactive effects [8] including anti-inflammatory, antioxidant [9,10], and anti-cancer properties [11]. The 4′-hydroxyl substitution on the phenolic ring of naringenin is responsible for its robust antioxidant activity, enabling it to efficiently scavenge free radicals and neutralize oxidative stress [12].

Despite its therapeutic benefits, regorafenib use is associated with several major adverse effects including hepatotoxicity, hypertension, dermatological toxicity, and hemorrhage [13]. Concerning its effect on liver, it causes observed hyperbilirubinemia [12], liver dysfunction (AST/ALT increase) and hepatic necrosis [13]. In contrast, naringenin is considered relatively safe with almost no significant side effects at this concentration, and it exerts diverse biological activities and suitable bioavailability according to [14,15]. It has a variety of biological effects that are effective against cancer, including cell growth inhibition, cell cycle arrest, and apoptosis induction. It also has antioxidant, anti-inflammatory, and hepatoprotective properties [12,14].

Accordingly, the present study was designed based on the hypothesis that combining naringenin with regorafenib could enhance anticancer efficacy while potentially mitigating regorafenib-associated adverse effects. Therefore, the IC_50_ of naringenin was combined with four different concentrations of regorafenib to determine the most effective treatment strategy, thereby giving the clinician the opportunity to reduce the regorafenib dose and hence its side effects.

Early detection of liver cancer remains challenging because of the lack of obvious symptoms and reliable diagnostic biomarkers. As a result, most patients with HCC develop metastasis with low survival rates [15].

E-cadherin gene is present on chromosome 16q22.1 [16]. It binds to the actin cytoskeleton to preserve the integrity of cell structure, prevent individual cells from moving, and assist in cell signal transmission. Aberrant expression of E-cadherin, which has a substantial impact on cell–cell communication, destroys the balance of epithelial tissues leading to the epithelial-barrier-integrity loss. This makes it easier for cells to become mobile and invasive, resulting in more vulnerable infiltration and metastasis [17,18].

Tetraspanins (TSPANs) are master organizers of the plasma membrane that are involved in intracellular trafficking, cell motility, and signal transduction [19].

Motility-related protein-1 [MRP1/Cluster of differentiation 9 (CD9)] is a member of TSPAN family, containing four transmembrane domains and two extracellular loops. It takes part in the formation of tetraspanin-enriched microdomains by its lateral connection with transmembrane proteins including integrins, proteases, and other adhesion proteins by altering the location and activity of these proteins [20]. Motility-related protein-1 has been shown to function either as a tumor suppressor or promoter relying on the type of tumor and the type of coupling protein associated with MRP1 gene [21,22].

The present study was performed to evaluate the inhibitory effect of regorafenib and naringenin solely or in combinations on the viability of HepG2 liver cancer cells and to assess their effect on E-cadherin and MRP1/CD9 gene expression.

## 2. Results

### 2.1. Inhibition of Cell Growth

**Figure 1:**
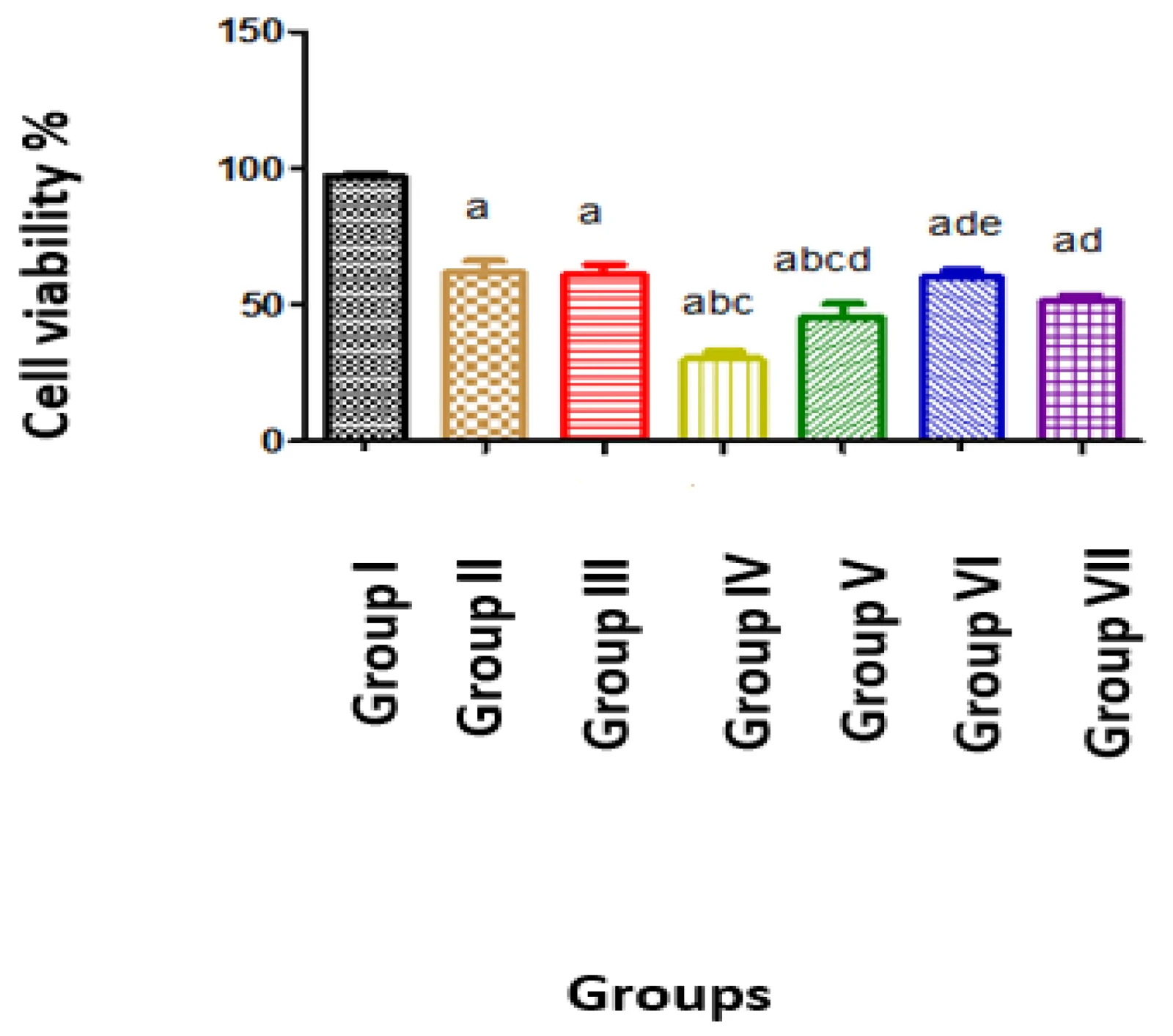
Cell viability of the studied groups. Group I: Untreated control, Group II: Naringenin-treated HCC (100 µM), Group III: Regorafenib-treated HCC (1 µM), Group IV: combination-treated group (Regorafenib 1 µM/Naringenin 100 µM), Group V: combination-treated group (Regorafenib 0.75 µM/Naringenin 100 µM), Group VI: combination-treated group (Regorafenib 0.5 µM/Naringenin 100 µM), Group VII: combination-treated group (Regorafenib 0.25 µM/Naringenin 100 µM). Data are expressed as mean ± standard deviation (SD) from three independent experiments (n = 3) performed in six technical replicates across seven experimental groups. The untreated control group was used as the calibrator and assigned a relative viability value of 1. Statistical analysis was performed using one-way ANOVA between all the studied independent groups and Student’s *t*-test between each two groups. The significant difference between individual groups was illustrated using small letters as follows: a: Significant difference from group I, b: Significant difference from group II, c: Significant difference from group III, d: Significant difference from group IV, e: Significant difference from group V at *p* ≤ 0.05.

Active growth of the HepG2 cells in the untreated control group (group I) was observed. Comparable levels of growth inhibition were observed in Groups II, III, VI, and VII. The greatest inhibition of growth was observed in group IV following treatment with the combination of naringenin and regorafenib at their respective IC50 (Figure 1).

### 2.2. E-Cadherin and MRP1/CD9 Gene Expression in the Studied Groups

**Figure 2:**
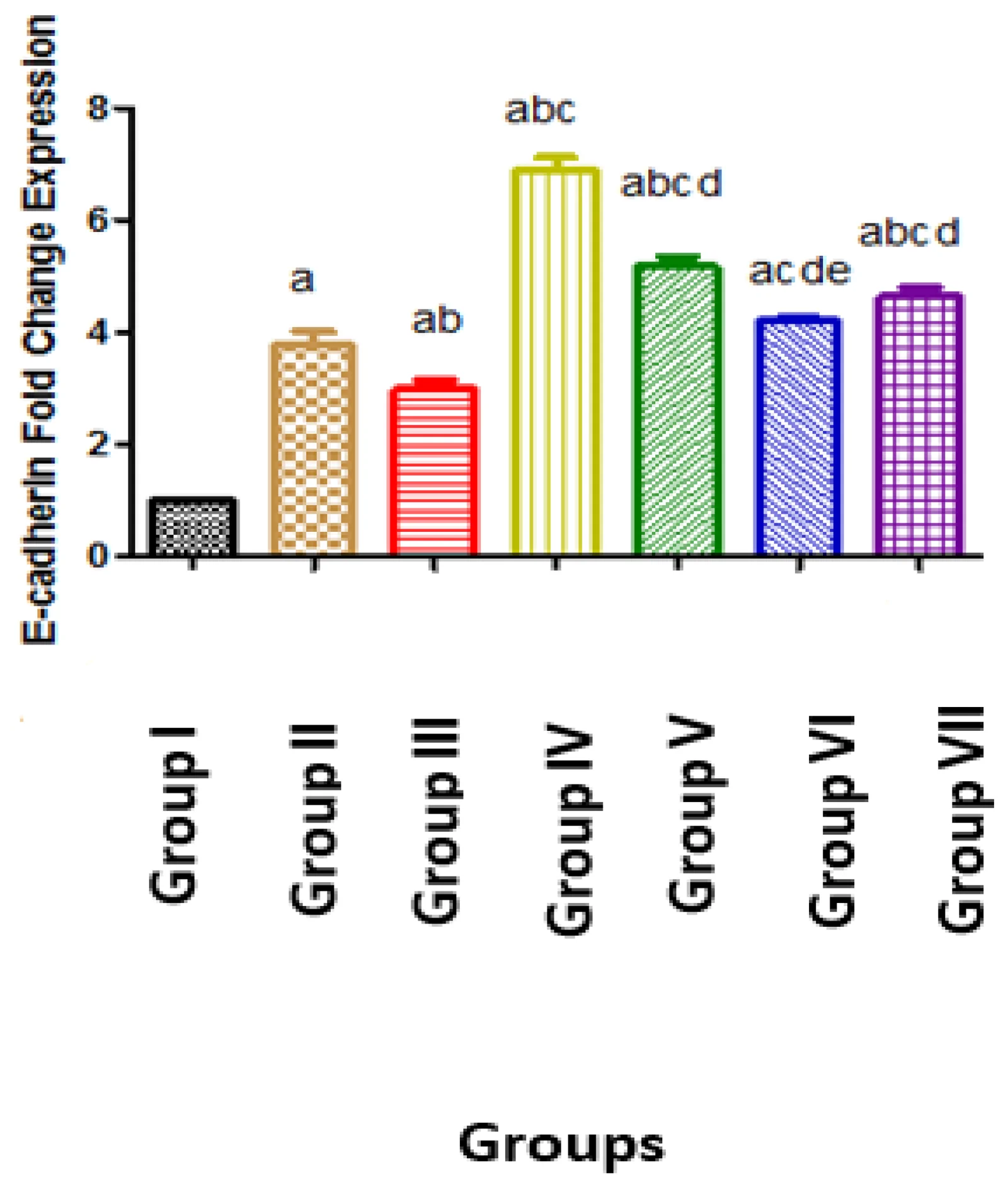
E-cadherin gene expression in the studied groups. Group I: Untreated control, Group II: Naringenin-treated HCC (100 µM), Group III: Regorafenib-treated HCC (1 µM), Group IV: combination-treated group (Regorafenib 1 µM/Naringenin 100 µM), Group V: combination-treated group (Regorafenib 0.75 µM/Naringenin 100 µM), Group VI: combination-treated group (Regorafenib 0.5 µM/Naringenin 100 µM), Group VII: combination-treated group (Regorafenib 0.25 µM/Naringenin 100 µM). Data are expressed as mean ± standard deviation (SD) from three independent experiments (n = 3) performed in six technical replicates across seven experimental groups. The untreated control group was used as the calibrator and assigned a relative *CDH1* gene expression value of 1.0. Statistical analysis was performed using one-way ANOVA between all the studied independent groups and Student’s *t*-test between each two groups. Significant difference between individual groups was illustrated using small letters as follows: a: Significant difference from group I, b: Significant difference from group II, c: Significant difference from group III, d: Significant difference from group IV, e: Significant difference from group V at *p* ≤ 0.05.

The expression of E-cadherin was upregulated by 3.78, 2.98, 6.9, 5.18, 4.20 and 4.65-fold in groups II, III, IV, V, VI, VII, respectively, compared with the untreated control group (group I) (Figure 2).

**Figure 3:**
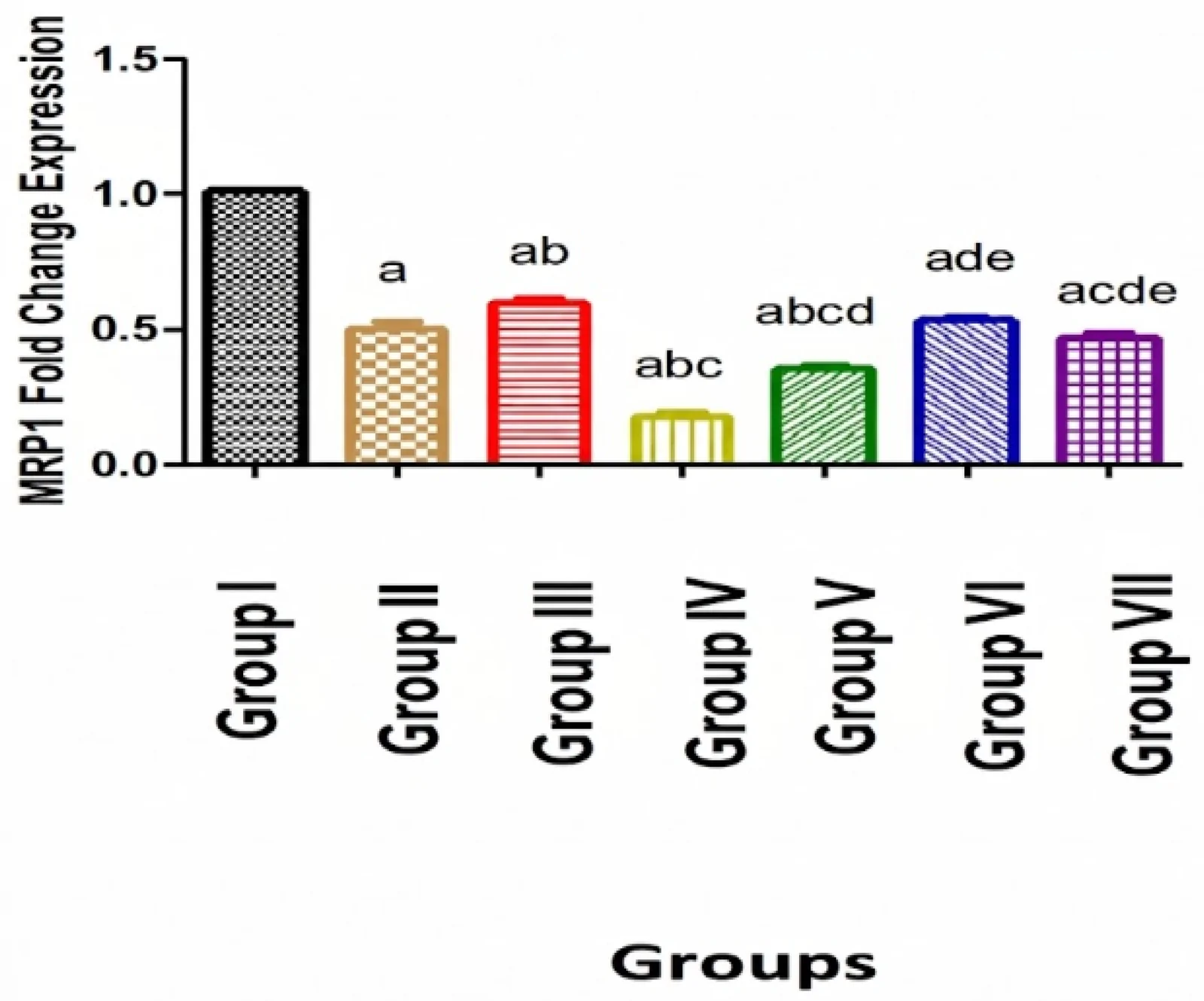
MRP1 gene expression in the studied groups. Group I: Untreated control, Group II: Naringenin-treated HCC (100 µM), Group III: Regorafenib-treated HCC (1 µM), Group IV: combination-treated group (Regorafenib 1 µM/Naringenin 100 µM), Group V: combination-treated group (Regorafenib 0.75 µM/Naringenin 100 µM), Group VI: combination-treated group (Regorafenib 0.5 µM/Naringenin 100µM), Group VII: combination-treated group (Regorafenib 0.25 µM/Naringenin 100µM). Data are expressed as mean ± standard deviation (SD) from three independent experiments (n = 3) performed in six technical replicates across seven experimental groups. The untreated control group was used as the calibrator and assigned a relative MRP1 gene expression value of 1.0. Statistical analysis was performed using one-way ANOVA between all the studied independent groups and Student’s *t*-test between each two groups. The significant difference between individual groups was illustrated using small letters as follows: a: Significant difference from group I, b: Significant difference from group II, c: Significant difference from group III, d: Significant difference from group IV, e: Significant difference from group V at *p* ≤ 0.05.

The level of MRP1 was downregulated by 0.50, 0.59, 0.17, 0.35, 0.53, 0.47-fold in groups II, III, IV, V, VI, VII, respectively, compared with group I. Each studied group showed significant differences with all other groups. The only non-significant differences were obtained between group II and each of group VI and VII (Figure 3).

### 2.3. Correlation Between the Studied Biomarkers in All Treated Groups

MRP1 and MTT had a strong positive correlation (r-value = 0.794, *p*-value < 0.0001), while strong negative correlations between E-cadherin and each of MTT and MRP1 were observed (r-value = −0.888, −0.738, respectively, and *p*-value < 0.0001 for each pair) (Table 1).

## 3. Discussion

Hepatocellular carcinoma (HCC) is the most common type of liver cancer and the third leading cause of cancer-related deaths worldwide [23].

In the current work, naringenin showed inhibition of growth of HepG2 cells in a dose-dependent manner in comparison to the untreated control group, with an approximate IC_50_ value of 100 µm (Figure 1).

These results are supported by previous studies. Pafumi et al. (2017) [24] explained that naringenin had anti-angiogenic effect in vitro and in vivo. They stated that naringenin impairs not only VEGF-induced vessel formation by impaired intracellular calcium signaling but also neovascularization in C57BL/6 mice. Moreover, Nasr Bouzaiene et al. (2016) [25] demonstrated that naringenin significantly suppressed the proliferation in B16F10 cells. This inhibitory effect was reported to be mediated by the suppression of DNA synthesis, induction of apoptosis and cell cycle arrest.

Qi et al. (2021) stated that Naringenin (20–160 μM) suppressed the cell migration and cell invasion tendencies of MDA-MB-231 breast cancer cells and explained this effect by apoptotic cell death via caspase activation [26].

Choi et al. [27] stated that the inhibitory action of naringenin on the growth of tumor cells is due to the ability to decrease phosphorylation of extracellular-regulated kinase (ERK) and phospho-c-Jun N-terminal kinase (JNK). Furthermore, naringenin treatment significantly promoted apoptosis via upregulation of the expression of activated caspase 3. Subsequently, caspase 3 promoted death-related signaling protein poly (ADP-ribose) polymerase inhibitor (PARP) cleavage and suppressed angiogenesis in malignant melanoma by affecting Tie-2 and angiopoietins-2 (Ang-2) expression.

In the current study, regorafenib had an estimated IC_50_ value of 1 µM and suppressed HCC cell growth in a dose-dependent manner in comparison to the untreated control group. This was in line with a study by Lin et al. [28] who found that regorafenib reduced the chemokine receptor (CXCR4) expression, hence decreasing the Wingless-related integration site (Wnt)/β-Catenin pathway transcriptional activity in gastric cancer cells, which subsequently inhibited the growth and invasion of gastric cancer cells.

Regarding combination groups, significant differences between each of groups IV, V, and VII and regorafenib-treated group (group III) were found in cell viability, with the greatest inhibition of cell viability observed in group IV as the addition of IC_50_ of both naringenin (100) µM and regorafenib (1 µM) dramatically limited cellular growth. Notably, the combination treatment retained enhanced antiproliferative activity even when the lowest concentration of regorafenib (0.25 μM) was used resulting in an undeniable suppression of cell proliferation and reduction in cell viability.

In the present work, E-cadherin and MRP1/CD9 were used to compare the different treatment regimens used in the study and to identify the most powerful “cellular migration/invasion inhibitor” treatment strategy from a molecular perspective. The present study’s results revealed that the use of naringenin and regorafenib was associated with a significant increase in the expression of E-cadherin and a significant decrease in the expression of MRP1 compared to positive control group. Interestingly, naringenin produced greater modulation of E-cadherin and MRP1 expression than regorafenib monotherapy (Figure 2 and Figure 3).

These findings corroborated those of recent research by Chen et al. and Martínez-Rodríguez et al. [29,30] who found that naringenin exhibited enhanced anticancer efficacy by enhancing E-cadherin expression while reducing cell invasion. It reduced the expression of vital molecules associated with the epithelial–mesenchymal transition (EMT) process, such as N-cadherin, vimentin, and Matrix metalloproteinase (MMP2/9). HAN et al. [31] found that exposing prostate cancer cells (PC-3) to 300 µM naringenin greatly increased E-cadherin expression and reversed EMT. They explained that by reducing the migration of PC-3 cells by suppressing urokinase plasminogen activator (UPA) activity, which has an important function in the invasion of cancer cells by degradation of the extracellular matrix.

Recent mechanistic insights emphasize that oxidative stress fuels cancer cell metastasis and invasive capacity through epigenetic mechanisms, notably by altering epigenetic landscape by recruiting and modulating the activity of epigenetic modifiers, thereby regulating the expression of metastasis-associated genes as E-cadherin [2]. The loss of E-cadherin promotes the invasion of tumor cells [2]. In our findings, treatment with naringenin upregulated E-cadherin. This therapeutic outcome can be partly attributed to naringenin’s well-established antioxidant efficacy [10], which neutralizes intracellular ROS levels and subsequently impedes ROS-induced epigenetic silencing of E-cadherin. By counteracting oxidative-driven gene silencing, the naringenin–regorafenib combination treatment could suppress EMT dynamics and offer a strategic mechanism to block oxidative-mediated metastatic signaling in HCC cells.

Regorafenib effect on cell invasion was explained by Zhang et al. [32] who stated that regorafenib markedly inhibits EMT in HCC cells via targeting inhibitor of differentiation (ID1), leading to cell migration, invasion suppression, and an increase in E-cadherin expression. Inhibitor of differentiation is a downstream target of bone morphogenetic protein 4 (BMP4) that stimulates EMT to induce cancer cell metastasis [33]. Chang et al. [34] explained that regorafenib inhibits fibroblast growth factor (FGF) and PDGF which are associated with yes-associated protein 1 (YAP1), leading to inhibition of EMT and cancer metastasis of cholangiocarcinoma, hence increasing E-cadherin level.

Fang and Kang [35] stated that E-cadherin loss has long been acknowledged as a feature of EMT, which is linked to a high risk of cellular motility and invasive potential. They explained how Snail, a key transcriptional factor involved in promoting EMT, regulates EMT by suppressing the production of E-cadherin. Their results are supported in the present study by the strong negative correlation between MTT and E-cadherin in all treated groups (r = −0.888, *p* < 0.0001) (Table 1).

Regarding the downregulation of the expression of MRP1 in naringenin- and regorafenib-treated groups compared to the untreated control group, this study agrees with a study by Beak et al. [36] who reported that increased MRP1 expression was linked to a poor outcome in individuals with acute lymphoblastic leukemia and breast cancer. Furthermore, Suhasini et al. [37] reported that MRP1 interacts with TGFα leading to consistent activation of epidermal growth factor receptor, thereby increases in cellular proliferation.

MRP1 has been shown in numerous cancer models to have pro-tumorigenic and pro-metastatic functions, despite previous description as a tumor suppressor. The coupling of MRP1 with different protein partners (Sumo-proteins) could explain its diverse roles in cancer cells [38]. Hence, it may be clinically significant to determine interactions of MRP1 in different cancer types that could impair cell motility [37].

Huan et al. [39] and Baek et al. [36] reported that MRP1 expression was linked to advanced tumor stages, cell motility, enhanced viability, and poor outcome in gastric and esophageal malignancies. Additionally, increased tumor cell invasiveness might be caused by the transcription of MMP2 triggered by MRP1 crosslinking, which in turn activates intracellular signaling molecules such as phosphatidylinositol 4-kinase (PI4K) and Src homology domain (Src homology 2). These results are supported in the present study by the strong positive correlation between MTT and MRP1 in all treated groups (r = 0.794, *p* < 0.0001) (Table 1).

The reduction in MRP1 gene expression following naringenin and regorafenib treatment is supported by Herr et al. [40] who explained that the treatment with 10 µM gefitinib, a drug from the same family of tyrosine kinase inhibitor as regorafenib, suppressed cell invasion. They explained that MMP breakdown of the surrounding extracellular matrix is the cause of cancer cell invasion. Motility-related protein-1 regulates pro-MMP-9 release in HT1080 cells and consequent cell invasion via EGFR release and activity. Therefore, inhibition of EGFR activity via tyrosine kinase inhibitors (as gefitinib) to suppress pro-MMP-9 production, reduces cellular invasion in MRP1-HT1080 cells via inhibiting EGFR activity [40].

Moreover, Fujiwara et al. [41] reported that cetuximab, which is another tyrosine kinase inhibitor, inhibits EMT-initiating effect of the oral squamous cell carcinoma extracellular vesicles (OSCC)-EVs (usually contain TSPANs, including MRP1). They reported OSCC-EVs upregulated the vimentin level, indicating that OSCC-EVs initiated EMT [42]. Contrary to our findings, Dükel et al. [43] stated that MRP1 was increased in the SW620 cells after naringenin treatment. They explained that naringenin could reduce the expression of protein kinase c (PKC) which is linked to cell adhesion molecules (CAMs) modification [44,45].

In the current study, concerning combination groups, regorafenib in concentration 1µM/naringenin in concentration 100 µM exhibited the highest level of E-cadherin and the lowest level of MRP1. Moreover, levels of E-cadherin showed significant increase and levels of MRP1 showed significant decrease in group VII (0.25 µM) compared to group III (Regorafenib 1 µM) emphasizing the enhanced beneficial effect even with a quarter the IC_50_ of regorafenib.

In the present work, a strong negative correlation was observed between MRP1 and E-cadherin in all treated groups (r = −0.738, *p* < 0.0001) (Table 1). This is consistent with the observations reported by Fujiwara et al. who stated that E-cadherin was altered by the MRP1-positive EVs in epithelial cells, pointing to EMT stimulation by OSCC-EVs. They explained that EGFR was highly expressed in OSCC cells, subsequently promoting the secretion of MRP1-rich EVs. The OSCC-EGFR-EVs could enter into epithelial cells and convert these cells into mesenchymal cells to initiate EMT, thus, decreasing E-cadherin [37].

On the contrary, Garner et al. [46] stated that MRP1 knockdown cells drastically reduced the expression level of E-cadherin by 80%. Moreover, the aggressiveness of cancer cells following the downregulation of MRP1 may be attributed to its E-cadherin expression regulation.

Collectively, the combination of regorafenib and naringenin produced enhanced combined inhibitory effects on HepG2 proliferation and modulated potential metastasis-related markers’ gene expression compared to single treatments.

## 4. Materials and Methods

### 4.1. Materials

Human hepatocellular carcinoma cell line G2 (HepG2) cells were purchased from American Type Culture Collection (ATCC, Rockville, MD, USA). The medium used for cell culture was Roswell Park Memorial Institute medium (RPMI-1640). Phosphate-buffered saline (PBS), Penicillin/streptomycin mixture, and RPMI-1640 media were purchased from Lonza, Walkersville, MD, USA. Fetal bovine serum (FBS) was purchased from Gibco™, Thermo Fisher Scientific Waltham, MA, USA, EU-approved grade, origin: Brazil. MTT reagent was obtained from Bio-Techne, Minneapolis, MN, USA (Cat# 4890-25-01).

Naringenin was purchased from Sigma Chemical Co., St. Louis, MO, USA. Naringenin was prepared as a solution in dimethyl sulfoxide (DMSO), kept at −20 °C, and diluted as required in cell culture media. Regorafenib was purchased from Selleck Chemicals (Houston, TX, USA). It was dissolved in sterile DMSO and stored at −20 °C. Just prior to usage, the working solution was diluted with culture media.

Working solutions were diluted in culture medium to ensure that the final DMSO concentration did not exceed 0.1% (*v*/*v*) across all treatment groups. Control cells were treated with matched vehicle control (0.1% *v*/*v* DMSO alone).

### 4.2. Methods

#### 4.2.1. Cell Culture Maintenance

HepG2 cells were cultured in a sterile tissue 50 cm^2^ flask using a complete media that contained RPMI 1640 along with 10% FBS, antibiotics (100 U/ml penicillin and 100 μg/ml streptomycin) in 95% air, 5% CO_2_ at 37 °C.

#### 4.2.2. Cell Viability Assay

The viability of HepG2 cells was tested by MTT assay (Trevigen Inc., Gaithersburg, MD, USA). Principle: The MTT assay is a colorimetric assay used to measure cellular metabolic activity as an indicator of cell viability, proliferation and cytotoxicity. The method depends on the reduction of tetrazolium salts. The yellow tetrazolium salt 3-[4, 5-dimethylthiazol-2yl]-2, 5-diphenyl-tetrazolium bromide (MTT) is reduced by the action of dehydrogenase enzymes from metabolically active cells, to generate reducing equivalents such as NADH and NADPH. The resultant intracellular purple formazan can be solubilized and quantitated colorimetrically in microtiter plates using spectrophotometer at 570 nm [47].

Procedure: HepG2 cells were seeded into 96-well plates at a density of 10^4^ cells per well and allowed to adhere overnight. Cells were then treated with growth medium containing varying concentrations of naringenin (25, 50, 75, 100, 125, 150, 175, 200 μM) or regorafenib (0.25, 0.5, 0.75, 1, 2, 3, 4 μM) in triplicate for 24 and 48 h, respectively. Following treatment, the medium was removed and 10 μL of MTT reagent was added to each well. Plates were incubated at 37 °C for 2–4 h to allow formazan crystal formation. The crystals were dissolved in DMSO, and absorbance was measured at 570 nm using a microplate reader. Cell viability was expressed as a percentage relative to untreated controls. IC50 values were determined by plotting drug concentration against cell viability (%) to generate dose–response curves.

Cell viability was calculated according to the equation:

Cell viability (%) = (Absorbance of treated cells/Absorbance of control cells) × 100.

The IC50 values of regorafenib and naringenin were found to be 1 µM and 100 µM, respectively [48,49,50].

Different concentrations of regorafenib were evaluated in combination with the IC50 of naringenin. Naringenin concentration was maintained at 100 µM [50] because it is considered relatively safe at this concentration while retaining its biological activity and bioavailability [51,52]. Hence, the present study aimed to preserve the full beneficial therapeutic effects of naringenin while reducing regorafenib concentration to potentially minimize its dose-related adverse effects [53,54].

#### 4.2.3. Determination of E-Cadherin and MRP1 Gene Expression in HepG2 Cells by RT-qPCR

Seven groups of HepG2 liver cancer cells were used, and each group was repeated six times; Group I: Untreated control group (Untreated HCC cells), Positive control was used because no normal cell line were used as negative control, Group II: Naringenin-treated HCC cells (100 µM incubated for 24 h), Group III: Regorafenib-treated HCC cells (1 µM incubated for 48 h), in addition to four Naringenin/Regorafenib combination groups with the following concentrations: Group IV: Regorafenib 1 µM/Naringenin 100 µM (incubated for 24 h), Group V: Regorafenib 0.75 µM/Naringenin 100 µM (incubated for 24 h), Group VI: Regorafenib 0.5 µM/Naringenin 100 µM (incubated for 24 h), Group VII: Regorafenib 0.25 µM/Naringenin 100 µM (incubated for 24 h).

The same number of HepG2 cells were seeded into different T25 flasks and incubated overnight for attachment. After 24 h, media were discarded and fresh media for control flasks were added. In the remaining flasks, fresh media containing the planned drugs’ concentrations were added according to the experimental design. After an incubation period of 24 h for naringenin [50], 48 h for regorafenib [49], and 24 h for combination groups, and after removing the culture medium, cells were trypsinized for 3 min at 37 °C and collected. Each experimental condition was performed using six independent biological replicates across three independent experiments. Storage of the cell pellets was carried out at −80 °C until extraction of Ribonucleic acid (RNA).

#### 4.2.4. Selection of Durations of Incubation for the Studied Groups

A 48 h treatment duration was selected for regorafenib monotherapy as supported by previous studies [49,55,56]. They found that a minimum exposure duration of 48 h was required to achieve substantial cell growth inhibition, whereas a 24 h exposure exhibited only minimal inhibitory effects. Regarding naringenin monotherapy, a 24 h incubation window was selected based on a previous study [50] who reported that the half-maximal inhibitory concentration IC50 of naringenin in human hepatocellular carcinoma (HepG2) cells was approximately 100 µM following a 24 h incubation period.

For the regorafenib/naringenin combination groups, a 24 h exposure was selected to evaluate the early synergistic effects of naringenin and to avoid excessive cell death, which would otherwise affect molecular assays (e.g., RNA extraction and qPCR analysis).

#### 4.2.5. Real-Time Quantitative Polymerase Chain Reaction (RT-qPCR) for Determination of Gene Expression of E-Cadherin and MRP1 in HepG2 Cells

Following the manufacturer’s instructions, RNA was extracted using the total RNA extraction kit (GF-1 TR kit, Vivantis Technologies, Subang Jaya, Malaysia). The concentration and purity of RNA yield was determined by measuring the absorbance at wavelength 260 (A_260_) and 280 (A_280_). The purity was obtained by dividing A260 to A280. The accepted ratio for A260/A280 is 1.9–2.1 for RNA. First, the cells’ whole RNA was extracted. Reverse transcription was performed to create complementary DNA (cDNA) using the extracted RNA as a template in a total reaction volume of 20 µL (Fermentas, Hanover, MD USA), which was then amplified using RT-qPCR in a total reaction volume of 25 µL per well (Thermofisher Scientific, Waltham, MA, USA).

Amplification and analysis of the RT-qPCR reaction data were carried out using Applied Biosystem’s software version 3.1 (StepOne^TM^, Thermo Fisher Scientific, Waltham, MA, USA). At the annealing temperature, the RT-qPCR assay with the primer sets was optimized. The PCR reaction was carried out as follows: one cycle for pre-denaturation of 50 °C for 2 min and 40 cycles of 95 °C for 15 s, 60 °C for 1 min, and 72 °C for 1 min. The primer efficiencies for E-cadherin, *MRP1*, and B-actin were adapted from previously validated studies. The gene bank accession number and the sequences of the primers used in the present study are described in Table 2.

Calculation of Relative Quantification (RQ) (relative expression):

The data were presented as cycle threshold (Ct). Ct values of the assessed genes (for E-cadherin, MRP1) and the housekeeping (reference) gene (β-actin gene) were included in the PCR data sheet [57] to normalize gene expression data across samples as a reliable internal control for E-cadherin and MRP1, demonstrating stable and consistent expression. Additionally, cellular mRNA expression levels of β-actin between controls and sample sets showed no notable differences, indicating that β-actin was a suitable reference gene [58,59,60].

Relative Quantification was calculated according to the standard Livak 2^−ΔΔCt^ method.

Step 1: The cycle threshold (Ct) values of the target genes were normalized to the reference gene (β-actin) for each individual sample to obtain Delta Ct values:

ΔCt = Ct target gene − Ct reference gene

Step 2: The ΔΔCt value for each sample was calculated by subtracting the mean ΔCt of the untreated control group (calibrator) from the ΔCt of the corresponding treated sample.

ΔΔCt = ΔCt_sample_ − ΔCt_control group_

Step 3: The relative gene expression (fold change, RQ) was finally calculated using the equation:

RQ (fold change) = 2^−(ΔΔCt)^

#### 4.2.6. Statistical Methods

Experimental Replicates: All assays and gene expression analyses were performed using independent biological replicates. In the present study, distinct cell cultures were seeded, treated, and processed separately; all viability assays and gene expression analyses were performed using independent biological replicates to ensure robust statistical power. Technical replicates involve repeated measurements or assays performed on the same biological sample (e.g., running the identical cDNA template during RT-qPCR). Data expresses results from three independent experiments (n = 3) performed in six replicates across seven experimental groups.

IBM SPSS statistics (V. 26.0, IBM Corp., Armonk, NY, USA, 2019) was used for data analysis. Data were expressed as mean ± SD for quantitative parametric measures. The following tests were done: data normality was first assessed using the Shapiro–Wilk test, and the data were found to be normally distributed; therefore, one-way analysis of variance (ANOVA) was used to evaluate the overall statistical variance across all the seven experimental groups simultaneously to explore whether significant differences existed among the groups for parametric data. Pairwise evaluations using Student’s *t*-test were performed to explore the statistical variance between each pair of the seven experimental groups. The probability of error ≤0.05 was considered significant, while ≤0.01 and 0.001 were considered highly significant.

## 5. Conclusions

The present study demonstrates that naringenin, a naturally occurring flavonoid with a favorable safety profile, enhances the anticancer activity of regorafenib in HepG2 cells. The combination treatment significantly reduced cell viability, increased E-cadherin expression, and decreased MRP1 expression compared with either agent alone. These findings suggest that naringenin may enhance the therapeutic efficacy of regorafenib and potentially enable the use of lower regorafenib concentrations, thereby reducing dose-related toxicity. However, these findings are limited to an in vitro model. Further studies, including formal drug-synergy analyses, migration and invasion assays, protein-level validation, animal studies, and clinical investigations, are required to confirm the therapeutic potential and translational relevance of this combination strategy.

## 6. Recommendations

1. Future in vivo studies using cancer animal models should investigate pharmacokinetics, pharmacodynamics, and drug metabolism to clarify anticancer activity, side effects, and optimal dosing/administration routes. Subsequent clinical trials are recommended, including evaluation of biochemical markers linked to cancer progression and metastasis.
2. Exploring the effect of naringenin in regorafenib-resistant HepG2 cells would highlight novel therapeutic opportunities.
3. Future work should include quantitative synergy calculations (e.g., Chou–Talalay method) in addition to colony formation assays followed by formal synergy quantification (e.g., Loewe additivity or Bliss independence methods) and analysis of ERK phosphorylation to strengthen mechanistic interpretation and evaluate combined treatment effect.
4. Functional validation through assays such as the in vitro scratch assay is warranted to confirm anti-migratory effects of the combination treatment. In addition, migration and invasion assays should be conducted to establish whether modulation of E-cadherin and MRP1 directly affects metastatic and invasive behavior in HCC cells.
5. Additional future investigations should include microscopic evaluation of morphology, flow cytometric analysis of cell cycle and apoptosis, and assessment of regulatory proteins. Wound healing and colony formation assays are also warranted to evaluate migration, proliferation, and clonogenic potential.
6. To further validate gene expression changes, future studies should incorporate protein-level validation specifically using Western blot analysis for key targets including E-cadherin, MRP1, and additional epithelial–mesenchymal transition (EMT) markers.
7. Although HepG2 is a well-established model, validating these findings across additional HCC lines (e.g., Huh7 or Hep3B) remains necessary in future studies.
8. Future studies incorporating non-cancerous liver cell models (such as primary human hepatocytes) are recommended to confirm the safety profile and selective cytotoxicity of regorafenib when used in lower doses.

## 7. Limitations

Synergy quantification using quantitative drug interaction assessments (e.g., Chou–Talalay combination index) was not performed. While the findings suggest synergism especially from molecular perspective, detailed analyses are needed to precisely strengthen mechanistic interpretation.

## Figures and Tables

**Table 1:** Correlation between the studied biomarkers in all treated groups.

Type of Correlation	r-Value	*p*-Value
Correlation between E-cadherin and MTT	−0.888	<0.0001
Correlation between MRP1 and MTT	0.794	<0.0001
Correlation between E-cadherin and MRP1	−0.738	<0.0001

The table represents *p*-values and r-values indicating the type of correlation. A strong negative correlation was observed between E-cadherin and MTT (r = −0.888), a positive correlation between MRP1 and MTT (r = 0.794), and a negative correlation between E-cadherin and MRP1 (r = −0.738). All correlations were statistically significant at (*p* < 0.0001).

**Table 2:** The gene bank accession number and the sequences of the primers used.

Gene Name	Gene Bank Accession Number	Primer Direction	Primer Sequence
E-cadherin (*CDH1*)	D49685	Forward	5′-GGTTATTCCTCCCATCAGCT-3′
		Reverse	5′-CTTGGCTGAGGATGGTGTA-3′
Motility Related Protein-1 (*MRP-1/CD9*)	X60111.1	Forward	5′-TGCATCTGTATC CAGCGCCA-3′
		Reverse	5′-CTCAGGGATGTAAGCTGACT-3′
β-actin gene (*ACTB*)	NM_001101.5	Forward	5′-AAGATCCTGACCGAGCGTGG-3′
		Reverse	5′CAGCACTGTGTTGGCATAGAGG-3′

## Data Availability

The original contributions presented in this study are included in the article. Further inquiries can be directed to the corresponding author.

## Abbreviations

Ang-2: Angiopoietin-2, ATCC: American Type Culture Collection, BRAF: B-Raf proto-oncogene, serine/threonine kinase, CD9: Cluster of differentiation 9, cDNA: complementary DNA, DMSO: Dimethyl sulfoxide, EGFR: Epidermal growth factor receptor, ERK: Extracellular signal-regulated kinase, EMT: Epithelial–mesenchymal transition, FBS: Fetal bovine serum, FGF: Fibroblast growth factor, FGFR: Fibroblast growth factor receptor, HCC: Hepatocellular carcinoma, HepG2: Human hepatocellular carcinoma cell line G2, HGF: Hepatocyte growth factor, HIF-1: hypoxia-inducible factor-1, IC_50_: Half maximal inhibitory concentration, ID1: Inhibitor of differentiation, JNK: c-Jun N-terminal kinase, MKI: Multi-kinase inhibitor, MMP: Matrix metalloproteinase, MRP1: Motility-related protein-1, MTT: 3-[4,5-dimethylthiazol-2yl]-2, 5-diphenyl-tetrazolium bromide, OSCC: Oral squamous cell carcinoma, PARP: Poly (ADP-ribose) polymerase, PC-3: Prostate cancer cells, PDGFRA: Platelet-derived growth factor receptor, alpha polypeptide, PDGFR-β: Platelet-derived growth factor receptor-β, PI4K: Phosphatidylinositol 4-kinase, PKC: Protein kinase C, RNA: Ribonucleic acid, RPMI: Roswell Park Memorial Institute medium 1640, RT-qPCR: Real-time quantitative polymerase chain reaction, SRC: Proto-oncogene tyrosine-protein kinase Src, TGF-β: Transforming growth factor-β Tie-2, Tie-2: Tyrosine kinase receptor-2, TSPAN: Tetraspanin, UPA: Urokinase plasminogen activator, VEGFR: Vascular endothelial growth factor receptor, Wnt: Wingless-related integration site, YAP1: Yes-associated protein.

## References

[1] Xu Y., Yu X., Sun Z., He Y., Guo W. (2022). Roles of LncRNAs Mediating Wnt/β-Catenin Signaling in HCC. Front. Oncol..

[2] Peng P., Qin S., Li L., He Z., Li B., Nice E.C., Zhou L., Lei Y. (2024). Epigenetic Remodeling under Oxidative Stress: Mechanisms Driving Tumor Metastasis. MedComm-Oncol..

[3] Cao X., Hou J., An Q., Assaraf Y.G., Wang X. (2020). Towards the Overcoming of Anticancer Drug Resistance Mediated by P53 Mutations. Drug Resist. Updat..

[4] Narayanan S., Cai C.Y., Assaraf Y.G., Guo H.Q., Cui Q., Wei L., Huang J.J., Ashby C.R., Chen Z.S. (2020). Targeting the Ubiquitin-Proteasome Pathway to Overcome Anti-Cancer Drug Resistance. Drug Resist. Updat..

[5] Wang B., Wang F., Ding A., Zhao H., Bu X. (2020). Regorafenib and Ginsenoside Combination Therapy: Inhibition of HepG2 Cell Growth through Modulating Survivin and Caspase-3 Gene Expression. Clin. Transl. Oncol..

[6] Khan K., Cascinu S., Cunningham D., Kim S.Y., Oki E., Seery T., Shen L., Siena S., Tournigand C., Turhal N.S. (2020). Imaging and Clinical Correlates with Regorafenib in Metastatic Colorectal Cancer. Cancer Treat. Rev..

[7] Miles E.A., Calder P.C. (2021). Effects of Citrus Fruit Juices and Their Bioactive Components on Inflammation and Immunity: A Narrative Review. Front. Immunol..

[8] Motallebi M., Bhia M., Rajani H.F., Bhia I., Tabarraei H., Mohammadkhani N., Pereira-Silva M., Kasaii M.S., Nouri-Majd S., Mueller A.-L. (2022). Naringenin: A Potential Flavonoid Phytochemical for Cancer Therapy. Life Sci..

[9] Flores-Peña R., Monroy-Ramirez H.C., Caloca-Camarena F., Arceo-Orozco S., Salto-Sevilla J.A., Galicia-Moreno M., Armendariz-Borunda J. (2025). Naringin and Naringenin in Liver Health: A Review of Molecular and Epigenetic Mechanisms and Emerging Therapeutic Strategies. Antioxidants.

[10] Dev A., Mumbrekar K.D. (2026). Naringenin Attenuates Cisplatin-Induced Hepatotoxicity and Nephrotoxicity by Restoring Glutathione Homeostasis and Suppressing Lipid Peroxidation in a Murine Model. bioRxiv.

[11] Qurtam A.A., Mechchate H., Es-Safi I., Al-Zharani M., Nasr F.A., Noman O.M., Aleissa M., Imtara H., Aleissa A.M., Bouhrim M. (2021). Citrus Flavanone Narirutin, In Vitro and In Silico Mechanistic Antidiabetic Potential. Pharmaceutics.

[12] Rashmi R., Bojan Magesh S., Mohanram Ramkumar K., Suryanarayanan S., Venkata SubbaRao M. (2018). Antioxidant Potential of Naringenin Helps to Protect Liver Tissue from Streptozotocin-Induced Damage. Rep. Biochem. Mol. Biol..

[13] Hou J.Y., Xiao Y.T., Huang J.B., Jiang X.H., Jiang K., Li X., Xu L., Chen M.S. (2022). Real-Life Experience of Regorafenib in Patients With Advanced Hepatocellular Carcinoma. Front. Pharmacol..

[14] Memariani Z., Abbas S.Q., ul Hassan S.S., Ahmadi A., Chabra A. (2021). Naringin and Naringenin as Anticancer Agents and Adjuvants in Cancer Combination Therapy: Efficacy and Molecular Mechanisms of Action, a Comprehensive Narrative Review. Pharmacol. Res..

[15] Hasan S., Jacob R., Manne U., Paluri R. (2019). Advances in Pancreatic Cancer Biomarkers. Oncol. Rev..

[16] Biswas K.H. (2020). Molecular Mobility-Mediated Regulation of E-Cadherin Adhesion. Trends Biochem. Sci..

[17] Bruner H.C., Derksen P.W.B. (2018). Loss of E-Cadherin-Dependent Cell–Cell Adhesion and the Development and Progression of Cancer. Cold Spring Harb. Perspect. Biol..

[18] Na T.-Y., Schecterson L., Mendonsa A.M., Gumbiner B.M. (2020). The Functional Activity of E-Cadherin Controls Tumor Cell Metastasis at Multiple Steps CELL BIOLOGY. Proc. Natl. Acad. Sci. USA.

[19] Florin L., Lang T. (2018). Tetraspanin Assemblies in Virus Infection. Front. Immunol..

[20] Reyes R., Cardeñes B., Machado-Pineda Y., Cabañas C. (2018). Tetraspanin CD9: A Key Regulator of Cell Adhesion in the Immune System. Front. Immunol..

[21] Li Y., Yu S., Li L., Chen J., Quan M., Li Q., Gao Y. (2020). KLF4-Mediated Upregulation of CD9 and CD81 Suppresses Hepatocellular Carcinoma Development via JNK Signaling. Cell Death Dis..

[22] Xing C., Xu W., Shi Y., Zhou B., Wu D., Liang B., Zhou Y., Gao S., Feng J. (2020). CD9 Knockdown Suppresses Cell Proliferation, Adhesion, Migration and Invasion, While Promoting Apoptosis and the Efficacy of Chemotherapeutic Drugs and Imatinib in Ph+ALL SUP-B15 Cells. Mol. Med. Rep..

[23] Amin N., Anwar J., Sulaiman A., Naumova N.N., Anwar N. (2025). Hepatocellular Carcinoma: A Comprehensive Review. Diseases.

[24] Pafumi I., Festa M., Papacci F., Lagostena L., Giunta C., Gutla V., Cornara L., Favia A., Palombi F., Gambale F. (2017). Naringenin Impairs Two-Pore Channel 2 Activity and Inhibits VEGF-Induced Angiogenesis. Sci. Rep..

[25] Nasr Bouzaiene N., Chaabane F., Sassi A., Chekir-Ghedira L., Ghedira K. (2016). Effect of Apigenin-7-Glucoside, Genkwanin and Naringenin on Tyrosinase Activity and Melanin Synthesis in B16F10 Melanoma Cells. Life Sci..

[26] Qi Z., Kong S., Zhao S., Tang Q. (2021). Naringenin Inhibits Human Breast Cancer Cells (MDA-MB-231) by Inducing Programmed Cell Death, Caspase Stimulation, G2/M Phase Cell Cycle Arrest and Suppresses Cancer Metastasis. Cell. Mol. Biol..

[27] Choi J., Lee D.H., Jang H., Park S.Y., Seol J.W. (2020). Naringenin Exerts Anticancer Effects by Inducing Tumor Cell Death and Inhibiting Angiogenesis in Malignant Melanoma. Int. J. Med. Sci..

[28] Lin X.L., Xu Q., Tang L., Sun L., Han T., Wang L.W., Xiao X.Y. (2017). Regorafenib Inhibited Gastric Cancer Cells Growth and Invasion via CXCR4 Activated Wnt Pathway. PLoS ONE.

[29] Chen Y.Y., Chang Y.M., Wang K.Y., Chen P.N., Hseu Y.C., Chen K.M., Yeh K.T., Chen C.J., Hsu L.S. (2019). Naringenin Inhibited Migration and Invasion of Glioblastoma Cells through Multiple Mechanisms. Environ. Toxicol..

[30] Martínez-Rodríguez O.P., González-Torres A., Álvarez-Salas L.M., Hernández-Sánchez H., García-Pérez B.E., Thompson-Bonilla M.d.R., Jaramillo-Flores M.E. (2020). Effect of Naringenin and Its Combination with Cisplatin in Cell Death, Proliferation and Invasion of Cervical Cancer Spheroids. RSC Adv..

[31] Han K.Y., Chen P.N., Hong M.C., Hseu Y.C., Chen K.M., Hsu L.S., Chen W.J. (2018). Naringenin Attenuated Prostate Cancer Invasion via Reversal of Epithelial–to–Mesenchymal Transition and Inhibited UPA Activity. Anticancer Res..

[32] Zhang N., Zhang S., Wu W., Lu W., Jiang M., Zheng N., Huang J., Wang L., Liu H., Zheng M. (2021). Regorafenib Inhibits Migration, Invasion, and Vasculogenic Mimicry of Hepatocellular Carcinoma via Targeting ID1-Mediated EMT. Mol. Carcinog..

[33] Deng G., Chen Y., Guo C., Yin L., Han Y., Li Y., Fu Y., Cai C., Shen H., Zeng S. (2020). BMP4 Promotes the Metastasis of Gastric Cancer by Inducing Epithelial-Mesenchymal Transition via ID1. J. Cell Sci..

[34] Chang Y.C., Li C.H., Chan M.H., Chen M.H., Yeh C.N., Hsiao M. (2022). Regorafenib Inhibits Epithelial-Mesenchymal Transition and Suppresses Cholangiocarcinoma Metastasis via YAP1-AREG Axis. Cell Death Dis..

[35] Fang C., Kang Y. (2021). E-Cadherin: Context-Dependent Functions of a Quintessential Epithelial Marker in Metastasis. Cancer Res..

[36] Baek J., Jang N., Choi J.E., Kim J.R., Bae Y.K. (2019). CD9 Expression in Tumor Cells Is Associated with Poor Prognosis in Patients with Invasive Lobular Carcinoma. J. Breast Cancer.

[37] Suhasini P.C., Shetty S.S., Kumari Nalilu S., Kumar Shetty P., Patil P. (2022). Tetraspanin CD9: A Friend or Foe of Head and Neck Cancer (Review). Oncol. Rep..

[38] Logozzi M., Di Raimo R., Mizzoni D., Fais S. (2020). Immunocapture-Based ELISA to Characterize and Quantify Exosomes in Both Cell Culture Supernatants and Body Fluids. Methods in Enzymology.

[39] Huan J., Gao Y., Xu J., Sheng W., Zhu W., Zhang S., Cao J., Ji J., Zhang L., Tian Y. (2015). Overexpression of CD9 Correlates with Tumor Stage and Lymph Node Metastasis in Esophageal Squamous Cell Carcinoma. Int. J. Clin. Exp. Pathol..

[40] Herr M.J., Kotha J., Hagedorn N., Smith B., Jennings L.K. (2013). Tetraspanin CD9 Promotes the Invasive Phenotype of Human Fibrosarcoma Cells via Upregulation of Matrix Metalloproteinase-9. PLoS ONE.

[41] Fujiwara T., Eguchi T., Sogawa C., Ono K., Murakami J., Ibaragi S., Asaumi J.I., Okamoto K., Calderwood S.K., Kozaki K.I. (2018). Anti-EGFR Antibody Cetuximab Is Secreted by Oral Squamous Cell Carcinoma and Alters EGF-Driven Mesenchymal Transition. Biochem. Biophys. Res. Commun..

[42] Fujita Y., Yoshioka Y., Ochiya T. (2016). Extracellular Vesicle Transfer of Cancer Pathogenic Components. Cancer Sci..

[43] Dükel M., Tavsan Z., Kayali H.A. (2021). Flavonoids Regulate Cell Death-Related Cellular Signaling via ROS in Human Colon Cancer Cells. Process Biochem..

[44] Abotaleb M., Samuel S.M., Varghese E., Varghese S., Kubatka P., Liskova A., Büsselberg D. (2018). Flavonoids in Cancer and Apoptosis. Cancers.

[45] Yan W., Yang J., Tang H., Xue L., Chen K., Wang L., Zhao M., Tang M., Peng A., Long C. (2019). Flavonoids from the Stems of Millettia Pachyloba Drake Mediate Cytotoxic Activity through Apoptosis and Autophagy in Cancer Cells. J. Adv. Res..

[46] Garner J.M., Herr M.J., Hodges K.B., Jennings L.K. (2016). The Utility of Tetraspanin CD9 as a Biomarker for Metastatic Clear Cell Renal Cell Carcinoma. Biochem. Biophys. Res. Commun..

[47] Garn H., Krause H., Enzmann V., Drößler K. (1994). An Improved MTT Assay Using the Electron-Coupling Agent Menadione. J. Immunol. Methods.

[48] Carr B.I., D’Alessandro R., Refolo M.G., Iacovazzi P.A., Lippolis C., Messa C., Cavallini A., Correale M., Di Carlo A. (2013). Effects of Low Concentrations of Regorafenib and Sorafenib on Human HCC Cell AFP, Migration, Invasion, and Growth In Vitro. J. Cell. Physiol..

[49] Carr B.I., Cavallini A., Lippolis C., D’Alessandro R., Messa C., Refolo M.G., Tafaro A. (2013). Fluoro-Sorafenib (Regorafenib) Effects on Hepatoma Cells: Growth Inhibition, Quiescence, and Recovery. J. Cell. Physiol..

[50] Arul D., Subramanian P. (2013). Naringenin (Citrus Flavonone) Induces Growth Inhibition, Cell Cycle Arrest and Apoptosis in Human Hepatocellular Carcinoma Cells. Pathol. Oncol. Res..

[51] Alzaabi M.M., Hamdy R., Ashmawy N.S., Hamoda A.M., Alkhayat F., Khademi N.N., Al Joud S.M.A., El-Keblawy A.A., Soliman S.S.M. (2022). Flavonoids Are Promising Safe Therapy against COVID-19. Phytochem. Rev..

[52] Zhang X., Li M., Wu H., Fan W., Zhang J., Su W., Wang Y., Li P. (2022). Naringenin Attenuates Inflammation, Apoptosis, and Ferroptosis in Silver Nanoparticle-Induced Lung Injury through a Mechanism Associated with Nrf2/HO-1 Axis: In Vitro and in Vivo Studies. Life Sci..

[53] Béchade D., Desjardin M., Castain C., Bernard P.H., Fonck M. (2017). Fatal Acute Liver Failure as a Consequence of Regorafenib Treatment in a Metastatic Colon Cancer. Case Rep. Oncol..

[54] Miners J.O., Chau N., Rowland A., Burns K., McKinnon R.A., Mackenzie P.I., Tucker G.T., Knights K.M., Kichenadasse G. (2017). Inhibition of Human UDP-Glucuronosyltransferase Enzymes by Lapatinib, Pazopanib, Regorafenib and Sorafenib: Implications for Hyperbilirubinemia. Biochem. Pharmacol..

[55] Fondevila F., Méndez-Blanco C., Fernández-Palanca P., González-Gallego J., Mauriz J.L. (2019). Anti-Tumoral Activity of Single and Combined Regorafenib Treatments in Preclinical Models of Liver and Gastrointestinal Cancers. Exp. Mol. Med..

[56] Alessandro R.D., Refolo M.G., Iacovazzi P.A., Pesole P.L., Messa C., Carr B.I. (2019). Ramucirumab and GSK1838705A Enhance the Inhibitory Effects of Low Concentration Sorafenib and Regorafenib Combination on HCC Cell Growth and Motility. Cancers.

[57] Livak K.J., Schmittgen T.D. (2001). Analysis of Relative Gene Expression Data Using Real-Time Quantitative PCR and the 2-ΔΔCT Method. Methods.

[58] Liu A.C., Gotlieb A.I. (2008). Transforming Growth Factor-β Regulates In Vitro Heart Valve Repair by Activated Valve Interstitial Cells. Am. J. Pathol..

[59] Pan Y., Li J., Zhang Y., Wang N., Liang H., Liu Y., Zhang C.Y., Zen K., Gu H. (2016). Slug-Upregulated MIR-221 Promotes Breast Cancer Progression through Suppressing E-Cadherin Expression. Sci. Rep..

[60] Hashida H., Takabayashi A., Tokuhara T., Hattori N., Taki T., Hasegawa H., Satoh S., Kobayashi N., Yamaoka Y., Miyake M. (2003). Clinical Significance of Transmembrane 4 Superfamily in Colon Cancer. Br. J. Cancer.

